# Psychoactive substance consumption after the Fundão dam mine tailing disaster in Minas Gerais State, Brazil

**DOI:** 10.1590/0102-311XEN237022

**Published:** 2024-03-11

**Authors:** Elaine Silva Miranda, Marcelo Dell'Aringa, Everaldo Alves da Costa, Thais Piazza, Francesco Della Corte, Luca Ragazzoni, Francesco Barone-Adesi, Carla Lourenço Tavares de Andrade, Claudia Garcia Serpa Osorio-de-Castro

**Affiliations:** 1 Faculdade de Farmácia, Universidade Federal Fluminense, Niterói, Brasil.; 2 Center for Research and Training in Disaster Medicine, Humanitarian Aid, and Global Health, Università del Piemonte Orientale, Novara, Italy.; 3 BlissMe Tech, Rio de Janeiro, Brasil.; 4 Faculdade de Medicina, Universidade Federal de Minas Gerais, Belo Horizonte, Brasil.; 5 Escola Nacional de Saúde Pública Sergio Arouca, Fundação Oswaldo Cruz, Rio de Janeiro, Brasil.

**Keywords:** Disasters, Drug Utilization, Pharmaceutical Services, Desastres, Uso de Medicamentos, Assistência Farmacêutica, Desastres, Utilización de Medicamentos, Servicios Farmacéuticos

## Abstract

Disasters cause changes in morbidity, mortality, and medicine use. Brazil is one of the main producers of mineral ores at great environmental cost. Mine tailings are stored in dams and ruptures have led to major disasters. We investigated the consumption of psychoactive medicines in the municipalities affected by the Fundão dam disaster in Minas Gerais State. An ecological study was carried out on drug consumption, estimated using public purchases in Minas Gerais and dispensing data from private retail pharmacies. Consumption (in number of defined daily doses/100,000 inhabitants per day) was analyzed descriptively in eight municipalities, stratified according to consumption level during a 25-month period. Six comparisons of mean consumption values for both data sets were done for pre- and post-disaster periods. The means of medicine consumption before and after the event were plotted and linear trends were added. Public purchase data evinced high consumption levels. Only pharmaceutical retail showed significant differences between the strata in the pre-disaster versus two post-disaster periods. Smaller municipalities showed an increase in consumption 15 months after the disaster. Clonazepam led the way in pharmaceutical retail consumption, followed by fluoxetine. Medicines showed an upward trend after the disaster. The high public provision may have stifled significant consumption patterns of psychoactive drugs; however, peak consumption were observed in private retail, suggesting a modification in use patterns after the disaster. The decrease in consumption immediately after the event was probably related to lower care-seeking behavior on the part of the population, and significant peaks after the disaster may reflect economic consequences of it.

## Resumo

Os desastres provocam alterações na morbidade, mortalidade e no uso de medicamentos. O Brasil é líder na produção de minérios com grande custo ambiental. Os rejeitos de mineração são armazenados em barragens e as rupturas dessas barragens têm causados grandes desastres. Investigamos o consumo de medicamentos psicoativos em municípios atingidos pelo desastre da Barragem de Fundão, em Minas Gerais. Foi realizado um estudo ecológico sobre o consumo de medicamentos, com base em dados de compras públicas e distribuição de farmácias privadas do varejo de Minas Gerais. O consumo (em número de doses diárias definidas/100 mil habitantes por dia) foi analisado descritivamente em oito municípios, estratificados segundo o nível de consumo durante um período de 25 meses. Foram feitas seis comparações de valores médios de consumo para os dois conjuntos de dados dos períodos pré- e pós-desastre. Foram calculadas as médias de consumo de medicamentos antes e depois do evento e adicionadas tendências lineares. Os dados de compras públicas mostraram elevados níveis de consumo. Apenas o varejo farmacêutico apresentou diferenças significativas entre os estratos no período pré-desastre versus dois períodos pós-desastre. Municípios menores apresentaram aumento no consumo a partir do 15º mês após o desastre. Clonazepam liderou o consumo no varejo farmacêutico, seguido pela fluoxetina. Os medicamentos apresentaram tendência de alta após o desastre. A elevada oferta pública pode ter afetado os padrões de consumo significativo de medicamentos psicoativos; no entanto, foram observados aumentos no comércio privado, sugerindo alterações nos padrões de uso após o desastre. A diminuição do consumo imediatamente após o evento estava provavelmente relacionada a um menor comportamento de procura de cuidados por parte da população e os aumentos significativos posteriores podem refletir as consequências econômicas do desastre.

## Resumen

Los desastres provocan cambios en la morbilidad, mortalidad y en el uso de medicamentos. Brasil es líder en la producción de minerales con grandes costos ambientales. Los desechos mineros se almacenan en represas y las roturas de dichas represas han causado grandes desastres. Investigamos el consumo de medicamentos psicoactivos en municipios afectados por el desastre de la presa de Fundão, en Minas Gerais. Se realizó un estudio ecológico sobre el consumo de medicamentos, con base en datos de compras públicas y distribución en farmacias privadas minoristas de Minas Gerais. El consumo (en número de dosis diarias definidas/100.000 habitantes por día) se analizó descriptivamente en ocho municipios, estratificados según el nivel de consumo durante un período de 25 meses. Se realizaron seis comparaciones de los valores medios de consumo para los dos conjuntos de datos de los períodos anterior y posterior al desastre. Se calculó el consumo medio de medicamentos antes y después del evento y se añadieron las tendencias lineales. Los datos de compras públicas mostraron altos niveles de consumo. Solo el comercio minorista farmacéutico presentó diferencias significativas entre los estratos en el período anterior al desastre frente a dos períodos posteriores al desastre. Los municipios más pequeños presentaron un aumento en el consumo a partir del 15º mes después del desastre. El clonazepam lideró el consumo en el comercio minorista farmacéutico, seguido de la fluoxetina. Los medicamentos presentaron una tendencia al alza después del desastre. La elevada oferta pública puede haber afectado los patrones de consumo significativo de medicamentos psicoactivos; sin embargo, se observaron aumentos en el comercio privado, lo que sugiere cambios en los patrones de uso después del desastre. La disminución del consumo inmediatamente después del evento probablemente relacionada con un menor comportamiento de búsqueda de cuidados por parte de la población, y los aumentos significativos posteriores pueden reflejar las consecuencias económicas del desastre.

## Introduction

Disasters of great magnitude have been increasing in Brazil and, in recent years, mining activity has contributed to the occurrence of two major events: the Mariana and the Brumadinho dam failures, both in the state of Minas Gerais. Municipalities in the state of Minas Gerais and Espírito Santo have faced several difficulties following the rupture of the Fundão dam in Mariana. On November 5, 2015, the collapse resulted in an estimated 34 million m^3^ of waste, water, and materials used in its construction, causing socioeconomic and environmental impacts in the Doce River basin. This event directly and indirectly affected at least 13 municipalities. The tailings crossed more than 650km to the mouth of the river in Linhares, on the coast of Espírito Santo ^1^.

In January 2019, 38 months later, another dam burst, this time at the Feijão Creek Mine, also in Minas Gerais, resulting in great loss of life, including more than 260 dead and 11 missing, considerable environmental and economic damage in 25 municipalities, directly affecting more than one million people ^2^. Hundreds of other dams remain at high risk of rupture throughout Brazil ^3^.

Disasters directly related to human action are precipitated by an abrupt event, built on an adverse context ^4^. The literature shows that the health consequences of disasters of this nature are considerable, including changes in morbidity, mortality, and drug use profile. The use of psychoactive drugs, especially, changes over time because of disasters. This change occurs both in the areas directly affected and in regions close to the site of the event ^5^ ^,^ ^6^.

This type of disaster is also usually associated with major psychological consequences compared to natural disasters with the same level of loss and damage. Events that occur as a result of human performance may subvert the feelings of trust and solidarity, considered essential for community life ^7^.

Based on these assumptions, the disaster caused by the Fundão dam burst is a relevant context for the consequences on mental health, including the use of psychoactive drugs ^8^. In events like this, the main indications for these medicines are frequently related to anxiety, depression, and posttraumatic stress ^8^. Increased drug consumption has been reported to occur after a 3-month lag phase ^9^, but this may be fleeting ^10^. Factors associated with an ineffective response may result in the affected population’s dependence on relief and remediation policies, which also play a role in the increased psychoactive drug consumption ^7^. Using drug utilization research (DUR) to monitor mental health outcomes related to drug consumption in this population may be a useful and ethical tool, since the studies may be replicated without directly approaching the affected population, avoiding the memory of the disaster and its negative consequences ^11^.

This study aimed to investigate whether there has been a change in psychoactive drug consumption in the municipalities directly affected by the rupture of the Fundão dam. Although a previous cross-sectional article studied psychoactive drug consumption in the municipality of Brumadinho ^12^, our approach is unprecedented in Brazil, and may be employed as a short- to long-term monitoring strategy after an event ^8^.

## Methods

### Study design and setting

We conducted an ecological study with two main data sources to estimate drug consumption, one from the public sector (drug purchases from the Minas Gerais State) and one from the private sector (all dispensing data from retail pharmacies). The assumption was that public purchases and private dispensing could be proxies for drug consumption ^1^ and could show differences in the profile before and after the November 2015 disaster.

Several municipalities were affected by the interruption of water supply as an aftermath of the Fundão dam collapse. Among them, we identified those located on the banks of the Doce River that held private pharmacy retail outlets. We considered that the environmental damage that caused the water shortage affected the livelihoods of the population, the generation of electricity, the suspension of industrial production, the interruption of commercial fishing, and damage to agriculture, livestock, and tourism. The following municipalities were affected: Alpercata, Belo Oriente, Galiléia, Governador Valadares, Itueta, Mariana, Resplendor, Tumiritinga, Aimorés, and Periquito.

### Selected medicines

Psychoactive drugs were selected based on a set of criteria, namely: (i) guidelines for the pharmacological treatment of anxiety disorders, obsessive-compulsive disorder and posttraumatic stress disorder ^13^; (ii) inclusion on the Brazilian List of Essential Medicines ^14^. Medicines used in mental health care are purchased by Brazilian municipalities. Supply is managed at the state level and distributed to primary health care services at the municipal level. Box 1 shows the 11 medicines selected.

**Box 1:** Selected psychoactive medicines.

NAME	DOSE	CLASS	ATC CODE	DDD *
Diazepam	5mg	Anxiolytics (benzodiazepine derivatives)	N05BA01	10mg
Midazolam	7.5mg/15mg	Hypnotics and sedatives (benzodiazepine derivatives)	N05CD08	15mg
Clonazepam	0.5mg/2mg	Antiepileptics (benzodiazepine derivatives)	N03AE01	8mg
Fluoxetine	10mg/20mg	Antidepressants (selective serotonin reuptake inhibitors)	N06AB03	20mg
Amitriptyline	25mg/75mg	Antidepressants (non-selective monoamine reuptake inhibitors)	N06AA09	75mg
Nortriptyline	25mg/75mg	Antidepressants (non-selective monoamine reuptake inhibitors)	N06AA10	75mg

ATC: Anatomical Therapeutic Classification; DDD: defined daily doses.

* Oral administration.

### Data sources

Public purchase data were obtained from the Integrated Management System for Pharmaceutical Services of the Minas Gerais State (SIGAF/MG, acronym in Portuguese), a centralized medicine acquisition system established in 2016, in which municipalities calculate demand, register, and adhere to state purchases and/or buy directly. The SIGAF/MG is a public procurement database with access granted under the *Brazilian Freedom of Information Act* (LAI, acronym in Portuguese). The system provided data on monthly municipal purchases of the same 11 medicines listed in Box 1.

Retail dispensing data from pharmacies originated from the Brazilian National System for the Management of Controlled Products (SNGPC, acronym in Portuguese). It enables the monitoring of controlled substances, including medicines such as psychoactive drugs and antimicrobials, in pharmacies and private drugstores. Information from the system includes drug name, dosage form and strength, and quantity dispensed in number of drug packaging units. This system is maintained by the Brazilian Health Regulatory Agency (Anvisa, acronym in Portuguese) and the data is now publicly available, although at the time the study begun, the data was obtained by the LAI and volume was limited.

Demographic information for each municipality per year was obtained from the Brazilian Institute of Geography and Statistics (IBGE, acronym in Portuguese).

### Analysis

Medicines were classified according to the World Health Organization (WHO) Anatomical Therapeutic Chemical (ATC) Classification System with their corresponding defined daily doses (DDD) ^11^. This classification is used worldwide to compare drug consumption and as a tool for the DUR. The DDD is a unit of measurement of consumption and does not refer to any specific dose-related indication.

Descriptive analyses focused on the 25-month period of public and private data, from July 2015 to July 2017. We standardized consumption by number of DDDs/100,000 inhabitants per day, using population estimates for each year of the period. Maximum and minimum values of private and public consumption of all medicines (in DDD/100,000 inhabitants/day) per municipality were calculated. Mean public and retail consumption were calculated for each municipality and each stratum over the study period.

The rationale for the analysis was the comparison of two independent groups with a limited number of observations, which indicates a nonparametric approach ^15^.

### Stratification and consumption proxy calculations

Because private consumption based on dispensing (and not purchases) may be a much more accurate indicator ^11^, pharmacy retail data was used to categorize municipalities into three different strata, estimated using nonconsecutive lower limits of consumption (Stratum 1: 9-14 DDDs/100,000 inhabitants/day; Stratum 2: 5-8.9 DDDs/100,000 inhabitants/day; Stratum 3: 0-4.9 DDDs/100,000 inhabitants/day). Public procurement data were then organized into the same strata. The organization into strata was done to allow for better observation of consumption during the study period.

For pharmaceutical retail data, we calculated the number of DDDs for each individual medicine dispensed per month and also calculated number of DDDs for all medicines.

For public consumption, we charted monthly purchases of each medicine in the affected municipalities. We assumed the quantity purchased in a given month would cover demand for the subsequent months until the next purchase and divided the quantity purchased by the number of months between purchases. We calculated the number of DDDs for each medicine per month. We added up the totals to produce the total monthly number of DDDs for all 11 medicines.

Additionally, we calculated the mean monthly pharmaceutical retail consumption in all municipalities for each medicine in DDDs/100,000 inhabitants/day for the 2015-2017 period.

### Graphs and statistical comparisons

The monthly mean consumption values in DDDs/100,000 inhabitants/day were calculated, including the municipal values in each stratum, from July 2015 to July 2017. Public and retail consumptions were plotted separately on monthly timelines (strata 1, 2, and 3). Four parameters were added to each graph: (i) a 4-month pre-disaster period (July-October 2015); (ii) the event (November 2015); (iii) a 4-month “lag period” ^9^ ^,^ ^16^ ^,^ ^17^, immediately after the event (December-March 2016); and (iv) three different 4-month periods, one for each stratum, in which the highest post-disaster consumption was visually observed and measured by total consumption in DDDs/100,000 inhabitants/day (June-September 2016 for Stratum 1; August-November 2016 for Stratum 2; and February-May 2017 for Stratum 3).

Differences between mean consumption for each stratum, in DDDs/100,000 inhabitants/day, were analyzed in two comparisons: pre-disaster (July-October 2015) versus immediate post-disaster (December-March 2016) for all three strata; and pre-disaster (July-October 2015) versus the three different post-disaster “peaks”.

Total consumption values for each medicine in all municipalities were calculated and the monthly means before and after the event were plotted and linear trends were added solely for visual description.

The Mann-Whitney test (Wilcoxon rank sum) method was used to compare pre- and post-disaster periods ^15^. The analysis was carried out using IBM SPSS, version 24 (https://www.ibm.com/), assuming a 5% significance level in the detection of differences between means. Tables and graphs, including linear trends, were created using Microsoft Excel (https://products.office.com/).

### Ethics statement

The data used in this study came from two different sources, one of which was publicly available (Anvisa). Access to the second data set (from the SIGAF/MG) was authorized and granted in accordance with the LAI. All information was unidentified and aggregated.

The study was submitted to Institutional Review Board of the Sergio Arouca National School of Public Health, Oswaldo Cruz Foundation, and received ethical waiver, in accordance with the exemption rule for non-identified aggregated public data in Brazilian ethical guidelines.

### Reporting

This study adhered to recommendations of the REporting of studies Conducted using Observational Routinely-collected health Data (RECORD) statement ^18^.

## Results

Data from Aimorés and Periquito (Minas Gerais) were not provided by Anvisa, so these municipalities were excluded from the analysis.

Table 1 shows maximum and minimum values for pharmaceutical retail and public purchases consumption of all medicines (in DDD/100,000 inhabitants/day) per municipality.

**Table 1:** Consumption ranges and means from pharmaceutical retail and public purchases for selected medicines in municipalities affected by the Fundão dam mine tailing disaster in Minas Gerais State, Brazil, 2015-2017, in defined daily doses/100,000 inhabitants per day.

Municipality	Private consumption * Range	Private consumption * Mean (SD)	Public consumption ** Range	Public consumption ** Mean (SD)
Stratum 1				
Mariana	12.82-48.20	18.65 (7.19)	32.32-359.30	191.03 (97.51)
Belo Oriente	9.28-54.16	24.18 (10.03)	0.63-136.92	73.54 (60.50)
Itueta	13.24-63.55	27.29 (10.95)	132.66-529.63	270.66 (102.00)
Stratum 2				
Governador Valadares	6.31-13.56	8.33 (1.65)	0.43-111.48	32.92 (39.02)
Resplendor	6.36-22.78	13.30 (5.20)	21.68-254.61	139.78 (65.71)
Stratum 3				
Alpercata	1.29-33.69	10.79 (8.33)	32.28-447.69	262.22 (115.03)
Tumiritinga	0.30-8.54	2.27 (2.15)	60.98-490.79	228.11 (137.77)
Galiléia	0.00-21.61	5.44 (5.66)	58.32-473.24	214.27 (106.66)

SD: standard deviation.

* Proxy based on pharmaceutical retail data;

** Proxy based on public purchases.

The maximum and minimal values show two main aspects: that the lower limit of pharmaceutical retail consumption defined the different strata, and that the maximum limits of public procurement data were high compared with their counterparts in pharmaceutical retail data, in which smaller municipalities (Stratum 3) have the lowest private values and the highest public consumption values.

The consumption means for pharmaceutical retail data in the 2015-2017 period were: 23.37 DDD/100,000 inhabitants/day for Stratum 1; 10.82 DDD/100,000 inhabitants/day for Stratum 2; and 6.17 DDD/100,000 inhabitants/day for Stratum 3. As for public consumption, Stratum 2 presented the lowest values, with an average of 86.35 DDD/100,000 inhabitants/day, followed by Stratum 1, with 178.41 DDD/100,000 inhabitants/day, and Stratum 3, with the highest values, 234.87 DDD/100,000 inhabitants/day.

**Figure 1:**
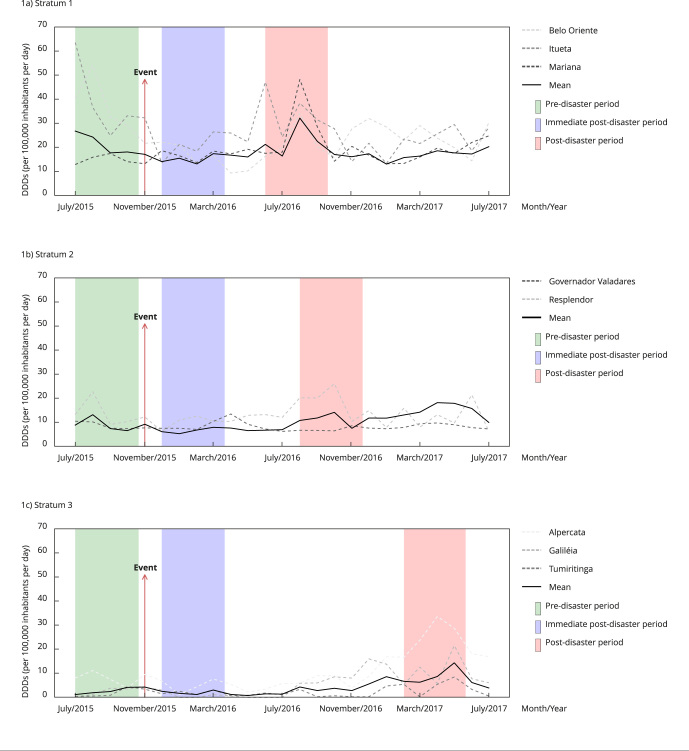
Monthly pharmacy retail consumption for selected medicines in municipalities affected by the Fundão dam mine tailing disaster in Minas Gerais State, Brazil, 2015-2017, in defined daily doses (DDD)/100,000 inhabitants per day.

**Figure 2:**
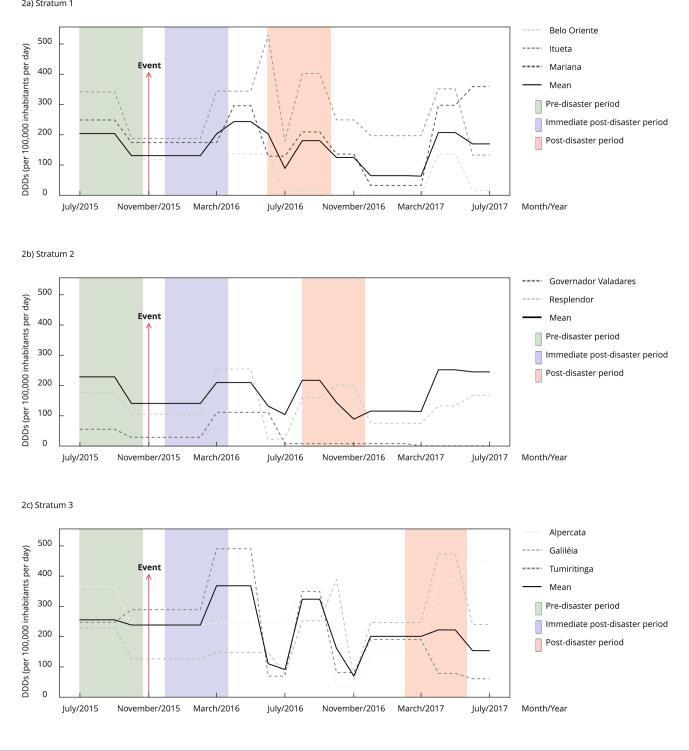
Monthly public purchase consumption for selected medicines in municipalities affected by the Fundão dam mine tailing disaster in Minas Gerais State, Brazil, 2015-2017, in defined daily doses (DDD)/100,000 inhabitants per day.

Figures 1 and 2 show monthly pharmaceutical retail consumption and public purchase consumption, respectively, in each stratum. The non-consecutive 4-month periods used for comparison purposes are highlighted in geometric forms (rectangles for the pre-disaster period, squares for the immediate post-disaster period, and rectangles for periods of peak consumption).

The statistical comparisons in Table 2 show that, of all the differences among the periods in each stratum and between pharmaceutical retail and public purchase consumption, the only significant results were obtained in the pre-disaster and the immediately post-disaster for Stratum 1 (p = 0.021) and for pre-disaster and post-disaster peak for Stratum 3 (p = 0.021).

**Table 2:** Comparison among pre-disaster, immediate post-disaster, and post-disaster highs consumption means (in defined daily doses/100,000 inhabitants per day) for pharmaceutical retail and public purchases of selected medicines according to strata in the municipalities affected by the Fundão dam mine tailing disaster in Minas Gerais State, Brazil, 2015-2017.

Period	Pharmaceutical retail (proxy for private consumption)			Public purchases (proxy for public consumption)		
	Monthly mean (SD)	1st comparison * p-value	2nd comparison ** p-value	Monthly mean (SD)	1st comparison * p-value	2nd comparison ** p-value
Stratum 1						
Pre-disaster ***						
July 2015	38.99 (25.40)	0.021	1.000	235.43 (113.76)	0.82	0.552
August 2015	35.53 (19.17)			235.43 (113.76)		
September 2015	24.97 (7.64)			235.43 (113.76)		
October 2015	25.33 (10.03)			160.07 (30.77)		
Immediate post-disaster ^#^						
December 2015	18.04 (4.35)			160.07 (30.77)		
January 2016	19.54 (2.64)			160.07 (30.77)		
February 2016	15.05 (2.84)			160.07 (30.77)		
March 2016	20.46 (5.20)			218.38 (110.46)		
Observed post-disaster peak in pharmaceutical retail ^##^						
June 2016	26.95 (17.47)			265.27 (228.97)		
July 2016	20.69 (3.23)			106.64 (86.46)		
August 2016	40.19 (7.26)			209.21 (193.30)		
September 2016	26.67 (5.78)			209.21 (193.30)		
Stratum 2						
Pre-disaster ***						
July 2015	11.94 (2.00)	0.564	0.386	116.73 (86.52)	0.429	0.231
August 2015	16.52 (8.85)			116.73 (86.52)		
September 2015	8.41 (1.10)			116.73 (86.52)		
October 2015	8.92 (2.07)			67.07 (54.21)		
Immediate post-disaster ^#^						
December 2015	6.98 (0.88)			67.07 (54.21)		
January 2016	9.3 (2.39)			67.07 (54.21)		
February 2016	9.9 (3.92)			67.07 (54.21)		
March 2016	10.5 (0.05)			183.05 (101.21)		
Observed post-disaster peak in pharmaceutical retail ^##^						
August 2016	13.53 (9.56)			83.77 (107.07)		
September 2016	13.47 (9.55)			83.77 (107.07)		
October 2016	16.33 (13.85)			104.67 (136.63)		
November 2016	9.56 (1.29)			104.67 (136.63)		
Stratum 3						
Pre-disaster ***						
July 2015	2.90 (4.50)	0.083	0.021	280.83 (73.88)	0.429	1.000
August 2015	4.38 (5.92)			280.83 (73.88)		
September 2015	4.10 (3.32)			280.83 (73.88)		
October 2015	4.03 (0.45)			218.46 (83.36)		
Immediate post-disaster ^#^						
December 2015	3.84 (2.75)			218.46 (83.36)		
January 2016	1.29 (1.30)			218.46 (83.36)		
February 2016	1.89 (2.29)			218.46 (83.36)		
March 2016	3.99 (3.34)			294.65 (176.74)		
Observed post-disaster peak in pharmaceutical retail ^##^						
February 2017	9.22 (6.53)			193.76 (51.63)		
March 2017	12.24 (11.74)			193.76 (51.63)		
April 2017	15.02 (16.17)			312.74 (207.42)		
May 2017	19.65 (10.28)			312.74 (207.42)		

SD: standard deviation.

Note: bold values for p < 0.05.

* Obtained from difference between period means (pre-disaster vs. immediate post-disaster);

** Obtained from difference between period means (pre-disaster vs. observed peak post-disaster);

*** A 4-month pre-disaster interval;

^#^ A 4-month “lag phase”, immediately after the event;

^##^ Three different 4-month periods, one for each stratum, in which the highest post-disaster consumption was visually observed and measured.

**Figure 3:**
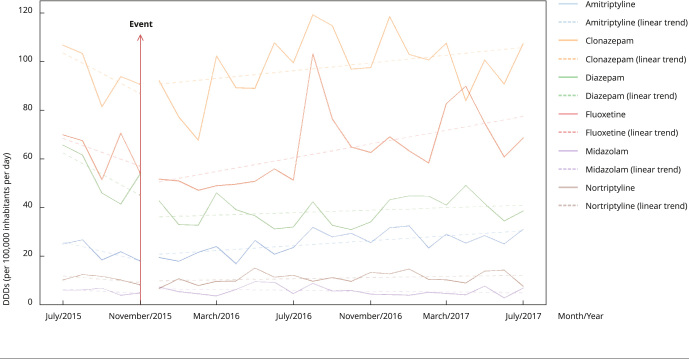
Linear trends in pre- and post-disaster pharmacy retail consumption for selected medicines in municipalities affected by the Fundão dam mine tailing disaster in Minas Gerais State, Brazil, 2015-2017.

Note: linear trends are given for the entire study period to show decrease or increase. Pre-disaster R^2^: clonazepam (0.364), fluoxetine (0.2423), diazepam (0.4579), amitriptyline (0.5603), nortriptyline (0.3691), midazolam (0.3594); pre-disaster angular coefficients: clonazepam (-2.84), fluoxetine (-2.87), diazepam (-4.38), amitriptyline (-1.46), nortriptyline (-0.64), midazolam (-4.38); post-disaster R^2^: clonazepam (0.1287), fluoxetine (0.3033), diazepam (0.071), amitriptyline (0.0472), nortriptyline (0.0832), midazolam (0.0607); post-disaster angular coefficients: clonazepam (0.80), fluoxetine (1.41), diazepam (0.25), amitriptyline (0.51), nortriptyline (0.12), midazolam (-0.08).

Clonazepam was the leader in pharmaceutical retail consumption, followed by fluoxetine, diazepam, and amitriptyline. Nortriptyline and midazolam were the least consumed. This balance remained the same for the entire period. However, individual medicines consumption showed apparent distinct linear trends before and after the disaster. Except for midazolam, which showed a downward trend during the entire period, clonazepam, fluoxetine, diazepam, nortriptyline, and amitriptyline showed an upward shift in pharmaceutical retail consumption from December 2015 onwards. Fluoxetine showed the largest angular coefficient for the linear trend equation in the post-disaster period, signaling a possible greater increase in consumption compared with the other psychoactive drugs (Figure 3).

Amitriptyline and fluoxetine showed reversed roles in pre- and post-disaster pharmaceutical retail consumption. The smallest fluctuations in consumption were shown in the pre-disaster period by amitriptyline and by fluoxetine in the post-disaster period (given by the highest R^2^ trend values), whereas the highest fluctuations in consumption (given by the lowest R^2^ value) were shown by fluoxetine in the pre-disaster period and by amitriptyline in the post-disaster period. Figure 3 shows the consumption of individual medicines over time.

## Discussion

This study compared pharmaceutical retail (private) and public purchases of antidepressants in eight municipalities affected by the Fundão mine tailing disaster. The main findings showed that, compared to private consumption, the public purchase proxy was much higher in volume, as shown in Table 2.

The public supply of psychoactive drugs was high throughout the 25-month study period, as can be seen from the range and mean consumption values, although there were drops in public consumption in Governador Valadares and Belo Oriente. The public purchasing data resulted in broad consumption ranges, with repeated values and plateaus, covering the demand of subsequent months until the next purchase ^19^. This is observed due to the purchasing system in Minas Gerais, in which state and municipal demands need to be grouped together in order to ensure economies of scale in purchases, implying an interval of two to three months between purchases.

Before arriving at the final three strata, several municipality were grouped to better determine the municipalities that best fit together, in terms of private consumption and limits. The choice of private consumption was based on the fact that dispensing data is a much more precise consumption proxy than purchase data ^11^, and the inferior limit was based on the reasoning that the disaster would unveil the increase and not the decrease in consumption. Furthermore, purchase data was linked to much higher values, not suitable for any limits. Stratum 1 showed “average” consumption ranges, both in private and public consumption. Stratum 3 showed a lower private consumption range and high public consumption, whereas Stratum 2 was similar to 3 in private consumption and similar to 1 in public consumption.

Figures 1 and 2 show how consumption progressed over time. The literature offers an insight about the possibilities for comparing drug consumption with the occurrence of a disaster: a pre-disaster period, an immediately post-disaster period, and a period, after the event, in which a consumption peak may be observed - and measured ^15^ ^,^ ^16^ ^,^ ^20^. However, although post-disaster peaks were observed, each study may follow a different pattern or consumption profile and may have employed an external comparison (another municipality), which did not occur in the Minas Gerais study. For this study we preferred a comparison between strata.

To better determine this possible profile, it was necessary to plot consumption over a 25-month period for each stratum. We also chose to represent the comparison periods of post-disaster peaks from pharmacy retail data by applying the same 4-month periods to public purchases.

In Brazil, the public sector is an important source of drug provision and medicines are provided free of charge. However, private expenditure on medicines is very high and 87.7% of household consumption of medicines is out-of-pocket ^21^.The high supply of psychoactive medicines in the public sector may have contributed to dissipate other observable peaks in private consumption. Nevertheless, the statistically significant results showed a diminished capacity for obtaining drugs from pharmaceutical retail in the first few months after the disaster, when there may be obstacles in accessing health services ^22^, or lower care-seeking behavior on the part of the population ^23^, or even immediate financial impacts resulting from the disaster ^24^. Municipalities within Stratum 1 included Mariana, where the event took place, possibly the hardest hit municipality, but, on the other hand, also the one in which post-disaster recovery funding was concentrated, thus reducing the medium- to long-term economic impact of the disaster to some degree ^24^.

The significant results in Stratum 3 were much more relevant because, although an increase was observed in pharmaceutical retail, a peak was also observed in public provision. If public provision had met needs, there would not have been a peak in pharmaceutical retail consumption. Thus, the need for psychoactive drugs 15 months after the event has greatly increased in these municipalities. This delayed response, coupled with intense consumption (compared with other strata), might be associated to belated economic difficulties as a consequence of the disaster. Data on other disasters show that long-term economic effects were only observed in the less affected municipalities - probably because of a lack of investments and disaster relief efforts ^24^.

According to IBGE averages for gross domestic product (GDP) per capita and the percentage of the population employed, Stratum 3 aggregates the smallest municipalities with the smallest economies. Strata 1 and 2 average population employment percentage was 19.3% and 18.05%, respectively, while Stratum 3 showed 8.43%. Regarding average GDP per capita, Stratum 1 showed BRL 34,057.2, followed by Stratum 2 with BRL 16.696,5, and Stratum 3 with BRL 9.327.03. Overall, in Minas Gerais, there was a reduction of BRL 96.62 billion in the state’s GDP, and it is estimated that some BRL 40.11 billion was caused by the disaster ^24^.

In all municipalities, the SNGPC-originated pharmaceutical retail data showed that clonazepam was the most consumed psychoactive drug, followed by fluoxetine, diazepam, and amitriptyline. This order did not change with the event and was the same throughout the study period.

Clonazepam is the most consumed psychoactive medicine in Brazil and its consumption increased 6-fold from 2009 to 2013. More than 2% of the adult population in the state of Rio de Janeiro uses clonazepam ^25^. Fluoxetine has been on the market for less time, but it is also the most widely used selective serotonin reuptake inhibitor (SSRI) in Brazil, the leader in sales and with widespread use, especially in older adults, as an “all-purpose” antidepressant ^26^. Notably, it also holds a safer metabolic risk profile than other antidepressants ^27^. In a 2016 study, chronic use of benzodiazepines was high in Brazilian state capitals ^28^ and Belo Horizonte, capital city of Minas Gerais, had the highest consumption of this class of medicines; diazepam consumption decreased, whereas other benzodiazepines, including clonazepam, showed increased consumption ^28^. A study from 2008 to 2012, carried out within the scope of primary health care in Ribeirão Preto, a large inland city in the state of São Paulo, showed that fluoxetine, amitriptyline, and clonazepam showed an increase in consumption over time ^29^. These previous consumption studies corroborate our results and also seem to validate the choice of medicines for this study.

Simple linear trends are not the best way to depict variation. However, the very low number of pre-disaster points prevented us from employing a more sophisticated approach ^30^. Nevertheless, our results are visually powerful, suggesting an apparent reversal of linear trends before and after the event. Apart from midazolam, all psychoactive drugs showed a shift from a decreasing trend before the event to an increasing trend (positive angular coefficient) after the disaster occurred, signaling a possible increase in consumption. Considering the increases in consumption diagnosed in the cited studies ^25^ ^,^ ^26^ ^,^ ^28^ ^,^ ^29^, it is also possible that the trends described are part of a greater overall increase in psychoactive drug consumption in the country. However, the contrasting downward trends before the event cast doubt on this explanation.

Previous work on disaster-related psychoactive drug consumption has shown a short-term and short-lived increase in prescriptions for sedatives and hypnotics immediately after the event. There was no observable impact on the consumption of antidepressant or antipsychotic three years after the event ^20^. Following the 2016 Sewol Ferry disaster in Ansan, South Korea, the number of prescriptions for antidepressants, anxiolytics, and sedative-hypnotics was compared between a case and a control municipality. A 6% increase in antidepressant prescription was observed in the affected municipality ^17^. Trifirò et al. ^16^ studied the use of antidepressants and antipsychotics before and after the L’Aquila earthquake and found an increase in the use of both medicines in older women two months after the event.

This study holds several limitations. The first is the dual source of consumption proxies - pharmaceutical retail and public purchases. Although these two sources are not comparable, they provide a reasonable context for the overall consumption of psychoactive medicines in the affected municipalities. It was necessary to assume a steady plateau for the consumption proxy based on purchasing data - even though they provide accurate volumes of state distribution to municipalities during the months of provision and reflect the predominant public nature of mental health drug provision in Brazilian municipalities ^18^.

On the other hand, municipalities may not only provide drugs purchased and distributed by the states. They can purchase individual medicines according to their municipal lists ^14^ and this would not be shown by the data. However, the municipalities involved in this study, except for Governador Valadares, are small and may not have sufficient budgetary resources to carry out a specific provision.

An underestimation of data may have occurred if any of the municipalities made a purchase and did not record it in SIGAF/MG, or if the company responsible for the mine and the disaster played a role in supplying medicines to the affected population.

Public municipal dispensing data was not available. The Brazilian Ministry of Health plans to link and make these data available in the future, but at the time of our study, only the states were sources of information, which was the case of Minas Gerais. Simultaneously, although the SNGPC data were very dense regarding the number of dispensations and retail outlets for each municipality, the dataset was short in width and only available for a limited number of months. This limited the number of data points to 25 (only four before the event), suggesting a recognized, albeit conservative dataset analysis ^31^ ^,^ ^32^, since the use of interrupted time-series analysis (ITS), jointpoint, or autoregressive integrated moving average (ARIMA) would produce a better fit with a larger number of data points before or after the truncated period ^33^ ^,^ ^34^. The differences-in-differences (DID) method would be ideal for a comparison of fluctuations before and after a disaster event, but our data does not comprise an “unexposed” group (a set of measurements parallel to the “exposed” data points) ^31^.

Finally, we were unable to produce age-related consumption data, to make better comparisons with the available national information on psychoactive consumption and with the international literature of drug consumption in disasters. This was the first study, to our knowledge, with a drug consumption approach based on large datasets linking increased psychoactive consumption to a major disaster in Brazil. Technological disasters may have long-term effects on mental health ^35^ ^,^ ^36^ and future research may clarify other patterns of morbidity we were unable to recognize in this study timeframe.

This study innovates by investigating not only pharmaceutical retail and public purchasing profiles in a disaster setting, but also by describing this phenomenon in different municipal strata, possibly linked to economic profile. Although consumption peaks were observed in all three strata, they occurred at different moments, perhaps signaling differences in the ability to cope with the disaster aftermath. The stratum with the smallest and poorest municipalities (3) may have been the one in which this effect was more intense.

The information generated by this paper aimed to guide the health system in future disasters and contribute to the rational use of medicines in disaster contexts. It may also guide the sectors involved in social and economic development to focus recovery strategies mainly on the most vulnerable municipalities.
